# The analysis of viability for mammalian cells treated at different temperatures and its application in cell shipment

**DOI:** 10.1371/journal.pone.0176120

**Published:** 2017-04-18

**Authors:** Juan Wang, Yun Wei, Shasha Zhao, Ying Zhou, Wei He, Yang Zhang, Wensheng Deng

**Affiliations:** 1 Institute of Biology and Medicine, Wuhan University of Science and Technology, Huangjiahu Campus, Wuhan, Hubei Province, China; 2 School of Chemistry and Chemical Engineering, Wuhan University of Science and Technology, Qingshan Campus, Wuhan, Hubei Province, China; EFS, FRANCE

## Abstract

Mammalian cells are very important experimental materials and widely used in biological and medical research fields. It is often required that mammalian cells are transported from one laboratory to another to meet with various researches. Conventional methods for cell shipment are laborious and costive despite of maintaining high viability. In this study we aimed to develop a simple and low-cost method for cell shipment by investigating the viabilities of different cell lines treated at different temperatures. We show that the viability of mammalian cells incubated at 1°C or 5°C significantly reduced when compared with that at 16°C or 22°C. Colony formation assays revealed that preservation of mammalian cells at 1°C or 5°C led to a poorer recovery than that at 16°C or 22°C. The data from proliferation and apoptotic assays confirmed that M2 cells could continue to proliferate at 16°C or 22°C, but massive death was caused by apoptosis at 1°C or 5°C. The morphology of mammalian cells treated under hypothermia showed little difference from that of the untreated cells. Quantitative RT-PCR and alkaline phosphatase staining confirmed that hypothermic treatment did not change the identity of mouse embryonic stem cells. A case study showed that mammalian cells directly suspended in culture medium were able to be shipped for long distance and maintained a high level of viability and recovery. Our findings not only broaden the understanding to the effect of hypothermia on the viability of mammalian cells, but also provide an alternative approach for cell shipment.

## Introduction

Mammalian cells including primary cells and cell lines are very important experimental materials and extensively utilized in the research field of biological and medical sciences. It is inevitable that the mammalian cells have to be shipped from one laboratory to another to meet with various researches around the world. Conventional method for cell shipment is that cryopreserved cells are transported with dry ice with in foam container; which shows little influence on cell features and maintains a high rate of cell viability [1]. However, cell shipment with dry ice is expensive and prohibited by the aviation departments of many countries [2]. An alternative method widely used by local companies or laboratories is directly to ship the cultured cells in the flask fully filled with cell culture medium [3, 4]; but the disadvantage of this method is not suitable for long-distance shipment [5]. Previous and recent studies showed that mammalian cells can be transported for long distance at ambient temperature by mixing the cells with agarose gel-or matrigel-based media [2, 6] and maintain a high rate of cell recovery after transportation for a few days. However, the procedures for these methods are complex and labor-consuming. Whether mammalian cells can be shipped in a simple mode at ambient temperature remains unclear.

Temperature is an important environmental factor for cell survival in vitro. Mammalian cells are usually cultured at 37°C in the incubator supplied with 5% of CO_2_ unless specific research purpose is required [7]. Previous studies showed that low temperature decreases cell growth rate and affects embryo development [8–10]; whereas mild heat stress enhances cell proliferation rate and accelerates development [11–12]. In addition, the viability for mammalian cells or embryos can be severely affected after long-term treatment at sub-zero temperature [13, 14]. It has been described that mononuclear cells were able to be obtained a better yield from whole blood cells shipped at environmental temperature of 22°C compared with the cells shipped at environmental temperature of 40°C [15]. Although the effect of temperature on cell viability has been studied for decades, the viability for mammalian cell lines directly suspended in their own culture medium and treated at different temperatures has not been systemically investigated. In this study, the viability of mammalian cell lines treated at different temperatures was analyzed at different time points. We show that M2 cells treated at 1°C or 5°C displayed low viability, whereas the cells treated at 16°C or 22°C maintained a considerable high viability. Colony formation assays revealed that M2 cells treated at 1°C or 5°C showed a poorer recovery compared with the cells treated at 16°C or 22°C. Similar results were obtained by analyzing other mammalian cell lines although their viabilities are distinct at 1°C or 5°C. A case study confirmed that mammalian cell lines could be shipped for long distance without severely affecting the viability.

## Materials and methods

### Cell culture

Melanoma cells (M2) were cultured in α-MEM complete medium (HyClone), HEK293T, Hela and mouse embryo fibroblast cells in high-glucose DMEM complete medium (HyClone), SaOS2 cells in Mc’coy 5A complete medium (HycClone), and K562 cells in RPMI 1640 complete medium (HyClone). Mouse embryo stem cells (mESC) were cultured in feeder-free condition, briefly, ES cells were cultured in knockout DMEM/F12 medium (Gibcol) containing 15% of knockout serum replacement, 0.1 mM non-essential amino acids, 2 mM L-glutamine and 0.05 mM 2-mercapitoethanol (Gibcol), 2 ng/mL human leukemia inhibitory factor (Sigma-Aldrich), 0.8 μM inhibitor PD0325901 (Selleck) and 3 μM inhibitor CHIR 99021 (Selleck). All cells used in the study were purchased from ATCC Co.

### Temperature treatment

Mammalian cells were trypsinized and harvested with a standard protocol. The cells were suspended with cell culture medium and diluted to the concentration of 1*10^5^ cells/mL. One milliliter of cell suspension were transferred into a 2-mL Eppendorf microtube and sealed with parafilm membrane. The cell samples were then treated at four different temperatures. Briefly, the sample for each cell line was divided into four groups and treated as follow: 1) Treatment at 1°C: samples were incubated in the ice-and-water mixture within a thermostatic container. The temperature inside container was measured and maintained at 1°C. 2) Treatment at 5°C: samples were incubated in the cold water that was pre-cooled and maintained at 5°C in a refrigerator (note that the actual temperature is 5°C measured by thermometer within refrigerator). 3) Treatment at 16°C: samples were incubated in the water bath that was maintained at 16°C. 4) Treatment at 22°C: samples were placed on the bench in the laboratory that was maintained at 22°C by air conditioner. The samples for each cell line were treated in triplicate at each temperature.

### Cell staining and viability analysis

Fluoresein diacetate (FDA) and propidium iodide (PI) stainings were used to determine live cells and dead cells respectively after hypothermic treatment. Basically, the live cells were stained into green color by FDA solution, the dead cells into red color by PI solution. Fluoresein diacetate and propidium iodide solutions were purchased from Sangon Biotech (Shanghai, China). Cell staining was performed with the method as recommended by the manufacturer’s manual. Briefly, after temperature treatment, the cells were spun down by centrifuge, the pellet was suspended in 500 μL of 1*PBS. 100 μL of the cells (approximately 2*10^4^ cells) were used to stain for 20 minutes by adding 50 μL of FDA and PI solution that contained 400 μg/mL of FDA and 5 mg/mL of PI. At the end of staining, the cells were washed for three times with 1*PBS, followed by suspending with 100μL 1*PBS and transferred into 96–well plate. The cells were precipitated by centrifugation at the speed of 218 g and imaged under fluorescent microscope (Olympus). To analyze viability, the stained cells including green and red cells were initially counted with flow cytometry (Beckman Coulter); the data were then used to statistical analysis. The viability was obtained by using the number of live cells divided by that of total cells. The value of the viability represents the mean of three independent experiments with standard deviation (SD).

### Colony formation assay

**Fig 1:**
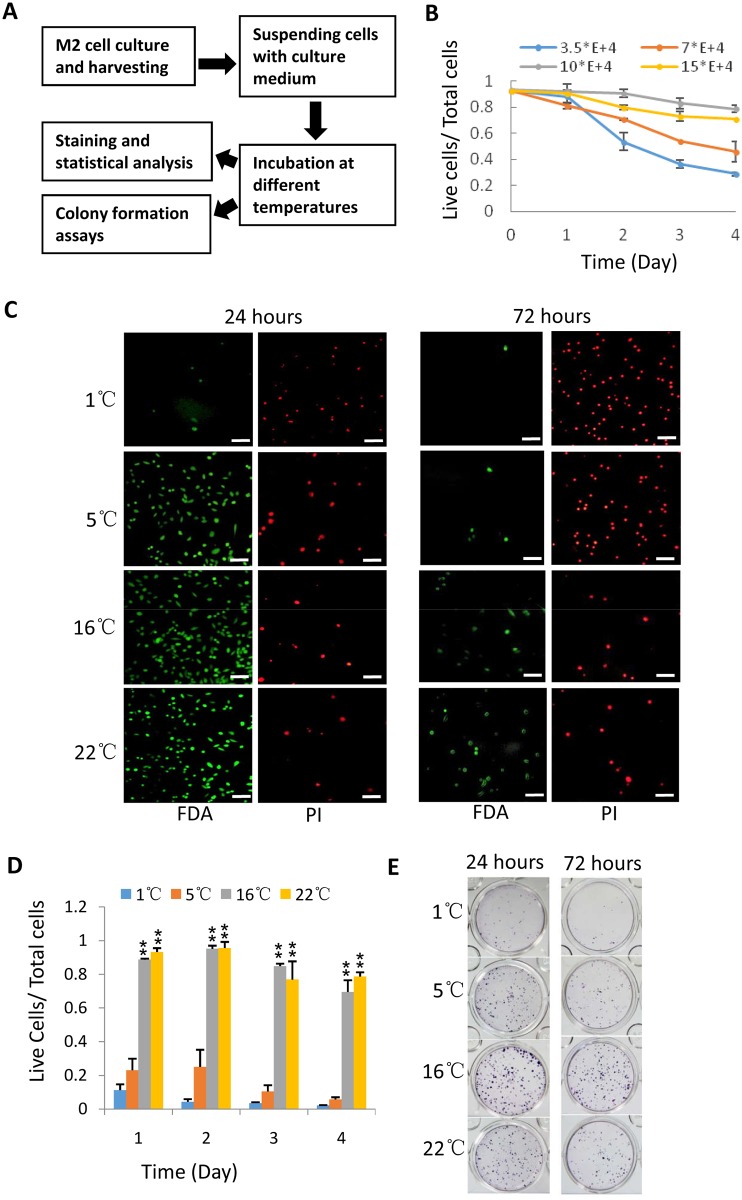
The treatment of severe hypothermia on M2 cells significantly reduced cell viability and recovery. A) A scheme for the procedure of cell treatment. M2 cells were cultured in 10-centimeter dish. When growing up to 70% of confluence, the cells were harvested and suspended in McCoy’s 5A complete medium, followed by incubation at different temperatures. Cell viability and recovery were analyzed at different time points using FDA and PI staining solution and colony formation assays. B) The analysis of viability for different concentrations of M2 cells incubated at 22°C. M2 cells were incubated at 22°C and stained by FDA and PI solution at different time points, the stained cells were counted with flow cytometry and analyzed for the viability. C) The analysis of fluorescence staining for M2 cells treated under hypothermia. M2 cells (1*10^5^) were incubated at indicated temperatures; the cells were stained with FDA and PI solution at different time points and imaged under fluorescence microscope. The scale bars represent 50 micrometer (μm). D) The effect of hypothermic treatment on the viability of M2 cells. M2 cells were treated and stained as in C. The viability was analyzed as in B. Each bar represents the mean of three independent experiments with standard deviation (SD). Significant difference was analyzed by comparing the viability at 1°C with that at other temperatures respectively. *represents P<0.05, ** represents P<0.01, P value was obtained by student’s *t* test. E) The analysis of colony formation for M2 cells treated under hypothermia. Colony formation assays were performed by using M2 cells treated at indicated temperatures for 24 hours or 72 hours. 800 of treated M2 cells were seeded in 12-well plate and cultured approximately for 10 days; cell colonies were used for Giemsa staining.

Colony formation assays were performed by seeding 800–1000 of cells in a 12-well plate depending on cell type (Figs 1 and 3). The cells were cultured for 10 days until cell colonies could be easily seen from the bottom of plate by naked eyes. For Giemsa staining, the cell colonies were rinsed with 1*PBS buffer, followed by fixation for 5 minutes with 4% of formaldehyde. After the fixation, the cell colonies were then washed for twice with 1*PBS buffer and stained with Giemsa solution (Sigma Aldrich) for 20 minutes at room temperature. The stained samples were then washed for 3 times with 1*PBS buffer,dried and pictured with digital camera (Cannon).

**Fig 2:**
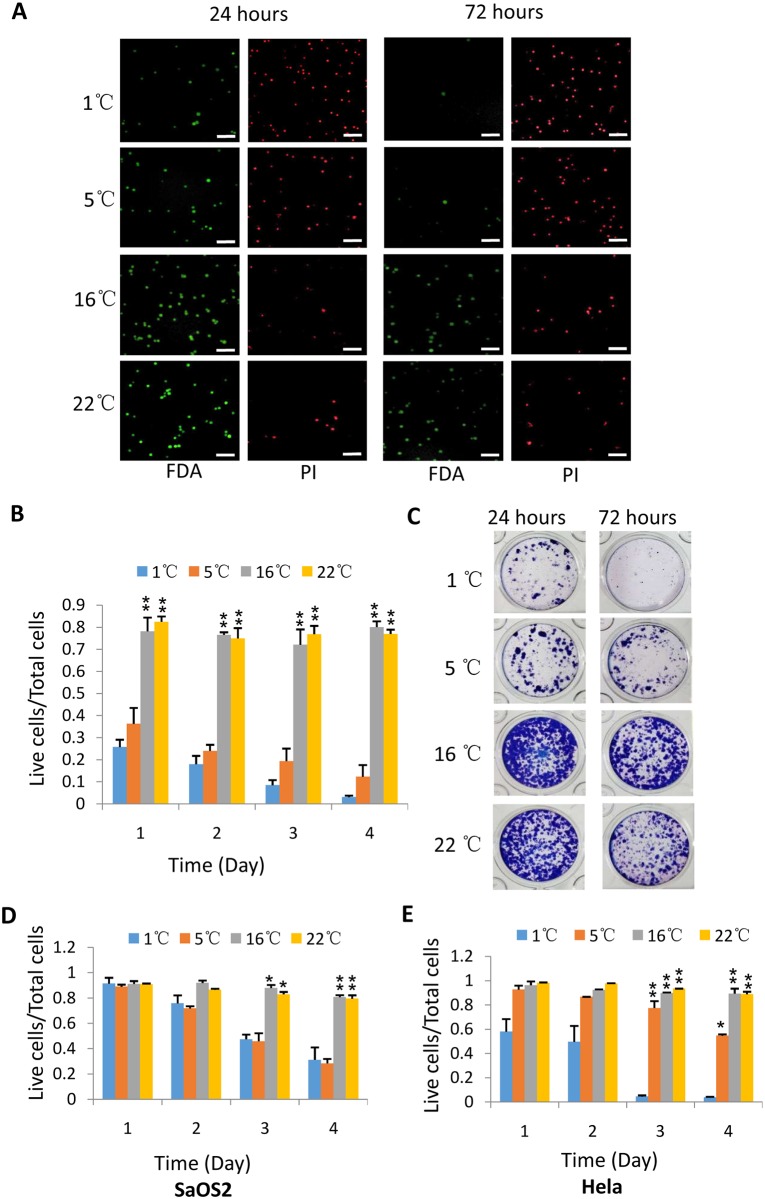
Hypothermic treatment differentially affects the viabilities of 293T, Saos2 and Hela cells. A) The analysis of fluorescence staining for 293T cells treated under hypothermia. 293 cells were stored at indicated temperatures, then stained and imaged as in Fig 1C. The scale bars represent 50 μm. B) Viability analysis for 293T cells incubated under hypothermia. C) The analysis of colony formation for 293T cells treated at indicated temperatures. Colony formation assays were performed as in Fig 1E. D) Viability analysis for SaOS2 cells. E) Viability analysis for Hela cells 293T, SaOS2 and Hela cells were treated and stained as in A. The viability was obtained as in Fig 1B. Each bar in B, D and E represents the mean of three independent experiments with standard deviation (SD). Significant difference between treatments was analyzed as in Fig 1D. *represents P<0.05, ** represents P<0.01, P value was obtained by student’s *t* test.

**Fig 3:**
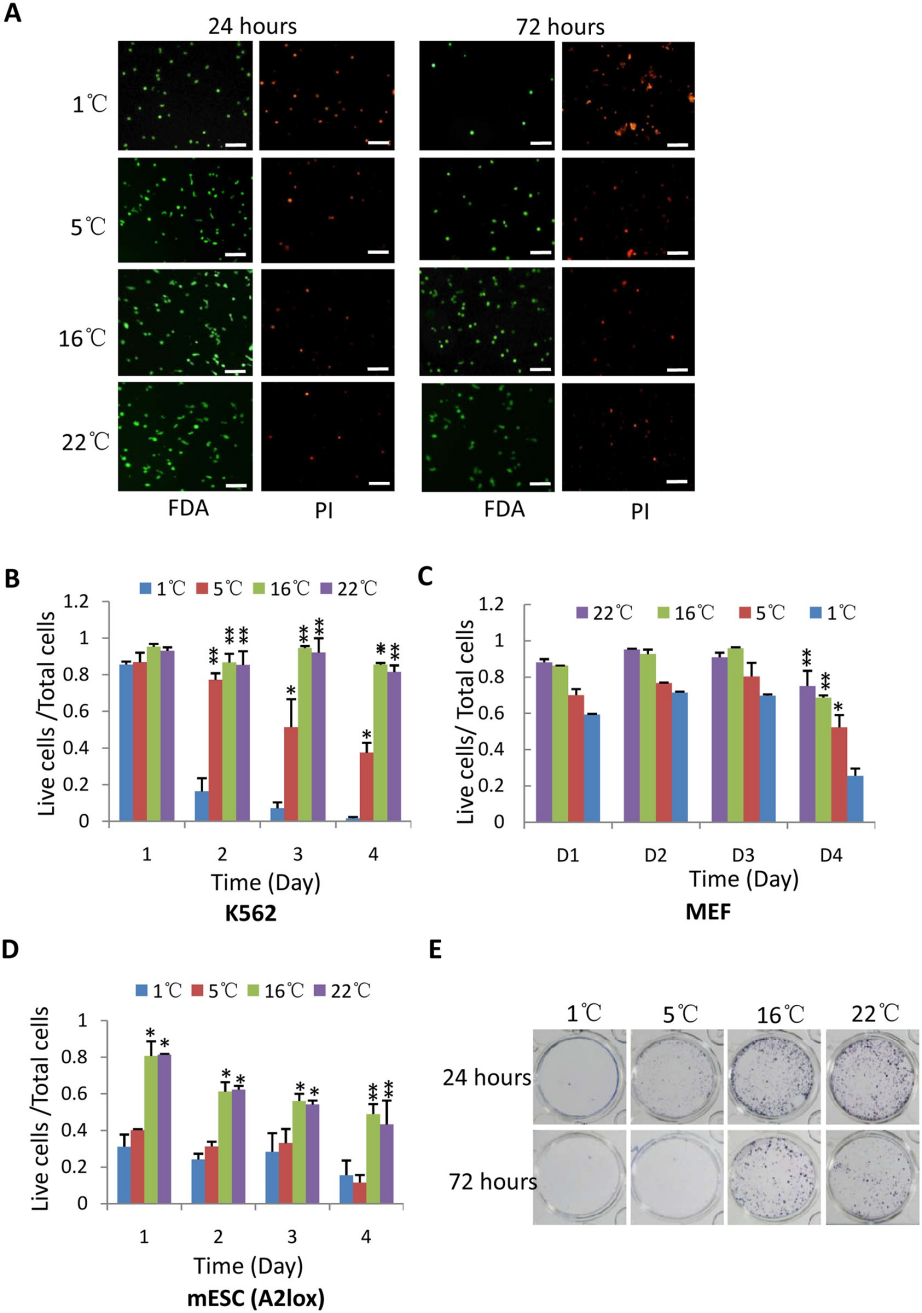
The effect of hypothermic treatment on the viabilities of suspension, primary and mouse ES cells. A) Analysis of fluorescence staining for K562 cells stored under hypothermia. K562 cells stored at indicated temperatures and stained at different time points as in Fig 1C. The scale bars represent 50 μm. B) The effect of hypothermia on the viability of K562 cells. K562 cells were treated and stained as in A. The viability was obtained as Fig 1B. C) The analysis of viability for MEF primary cells stored under hypothermia. The treated MEF cells were analyzed as in B. D) The effect of hypothermia on the viability of mouse ES cells. Stem cells were cultured as described above, viability were analyzed as in B. E) Colony formation analysis for mouse embryonic stem cells. 1000 of treated cells were seeded and cultured in 12-well plate. After growing 10 days, cell colonies were stained as in Fig 1E. Each bar in B-D represents the mean of three independent experiments with standard deviation (SD). Significant difference was analyzed as in Fig 1D. *represents P<0.05, ** represents P<0.01; P values were obtained by student *t* test.

### MTT assay

MTT (Methylthiazolyldiphenyl-tetrazolium bromide) powder (Sangon Biotech) was dissolved in 1*PBS(PH 7.4) to obtain the solution with a final concentration of 5 mg/mL. The solution was sterilized by filtration with a 0.22μm-membrane filter and preserved in a 50 mL tube wrapped with tin foil to prevent from the light. The treated cells (1*10^5^) were suspended in 1 mL medium by gentle pipetting at each time point, 50 μL of cell suspension was added to a 96-well plate, followed by adding 20 μL of MTT solution to each well. The cells were incubated at 37°C for 4 hours,150 μL of DMSO was added to each well. The samples were incubated at room temperature for 10 min with agitation on a rocker. The OD value was measured at the wavelength of 490 nm by SpectraMax i3 (Molecular Device).

### Apoptotic and necrotic assay

Apoptotic and necrotic detection kit was purchased from Sangon Biotech. Staining solution was prepared according to the manufacturer’s manual. M2 cells were treated at different temperatures as described above. 500μL of treated cells (0.5*10^5^) was collected by centrifugation at 1500 rpm for 5 min at room temperature; cell pellet was suspended and stained with 100μL of staining solution at room temperature for 45 min. The cells were then re-suspended with 100 μL of assay buffer and subjected to counting with flow cytometry.

### Alkline phosphatase staining and RT-qPCR

One thousand of mouse embryonic stem cells were seeded in 12-well plate and cultured in feeder-free condition. When the cells grew for 10 days, cell colonies were used for alkaline phosphatase staining. Briefly, cell colonies were fixed by 4% of formaldehyde that was prepared with 1*PBS buffer. When fixation finished, the cell colonies were washed for 3 times with 1*PBS buffer and stained for 1 hour with BCIP and NBT staining solution (Sangon, Biotech). The samples were washed for twice with 1*PBS buffer after staining and dried for photography. To perform RT-qPCR, the total RNA was prepared from mouse embryo stem cells and mouse embryonic fibroblast cells with RNA miniprep kit (Axygene). cDNA were synthesized with 0.5 μg of total RNA using 1 U of reverse transcriptase (Thermo). Quantitative PCR was performed with SYBR Green reaction mixture (Thermo) and Bio-Rad real time detection system. The qPCR data were analyzed by Bio-Rad CFX Manager 3.1 software. Relative gene expression was obtained by comparing the gene expression in stem cells with that in MEF, in which the gene expression in MEF was arbitrarily set as 1.

## Results

### The treatment of severe hypothermia on M2 cells significantly reduces cell viability and recovery

In this study we aimed to understand the effect of hypothermic treatment on the viability of mammalian cells and develop a simple approach for cell shipment. To achieve this goal, four types of temperature treatments were designed as detailed in Materials and Methods. A pilot experiment was initially performed with human melanoma cells (M2). 3*10^5^ of M2 cells were suspended with 1 mL of culture medium within a 2-mL Eppendorf microtube, followed by hypothermic treatment and stained by FDA and PI solution; the stained cells were then subjected to statistical analysis (Fig 1A). Unexpectedly, M2 cells incubated at 16°C or 22°C showed a higher viability than they at 1°C or 5°C (here 1°C and 5°C are designated as severe hypothermia, 16°C and 22°C as mild hypothernia); however, the viability of the cells incubated at 22°C showed instability (S1 Fig, Supporting Information). We supposed that the unstable result might be caused by the temperature and the concentration of M2 cells. Next, the cell concentration was optimized by diluting different number of cells in 1 mL of culture medium. The cells were incubated at 22°C; viability was analyzed at different time points. As shown in Fig 1B, the concentration of 1*10^5^ cells/mL displayed the highest viability among the tested concentrations. Therefore, the concentration of 1*10^5^ cells /mL was used for onward experiments in the study.

We next re-examined the effect of hypothermia on the survival of M2 cells using the optimal concentration. After hypothermic treatment, M2 cells were stained with FDA and PI solution and imaged under fluorescence microscope. Fig 1C shows that M2 cells incubated at 1°C or 5°C for 24 hours exhibited more dead cells (red) than they at 16°C or 22°C. When the time of incubation was prolonged to 72 hours, the difference for the number of dead or live cells between severe hypothermia and mild hypothermia became prominent (Fig 1C). The stained cells were then counted by flow cytometry and analyzed for the viability. The data showed that the viability of M2 cells treated at 1°C or 5°C significantly reduced compared with that at 16°C or 22°C (Fig 1D), indicating that severe hypothermia is more harmful to M2 cell survival *in vitro* than mild hypothermia. To verify the observation, colony formation assays were performed after incubation under hypothermia for 24 hours or 72 hours. As shown in Fig 1E, M2 cells incubated at 16°C or 22°C formed more colonies than they at 1°C or 5°C although the number of colonies slightly decreased when the time was prolonged. Together, these data indicate that severe hypothermia significantly damages the viability of M2 cells and their recovery; whereas mild hypothermia can improve the survival status of M2 cells.

### Hypothermic treatment differentially affects the viabilities of 293T, Saos2 and Hela cells

Our initial experiments revealed that M2 cell survival was severely affected by severe hypothermia. We next asked whether this observation can be extended to other types of cells. To answer the question, 293T, SaOs2 and Hela cells were used to investigate the effect of hypothermia on cell viability using the same approaches as in M2 cells. Fig 2A and 2B show that the viability of 293T cells significantly decreased when incubated at 1°C or 5°C compared with that at 16°C or 22°C. Colony formation assays confirmed that preservation of 293T cells at 1°C or 5°C led to a poorer recovery than that at 16°C or 22°C (Fig 2C), indicating that severe hypothermia not only damages the viability but also cell recovery. This observation is consistent with that obtained in M2 cells (Figs 1E and 2B). Intriguingly, the survival rate for SaOS2 cells remained little change when incubated at different temperatures for 24 hours, but it significantly reduced after incubation for 48 hours at 1°C or 5°C, particularly for the cells stored at 1°C (Fig 2D), suggesting that the endurance of SaOS2 cells to severe hypothermia is stronger than that of M2 or 293T cells. Fig 2E demonstrates that the survival rate of Hela cells did not show much difference between 5°C and 16°C or 22°C under incubation for 72 hours, indicating that Hela cells are less sensitive to the temperature of 5°C compared with M2 and 293T cells. However, Hela cells showed more death when stored at 1°C over 48 hours (Fig 2E). Taken together, these data indicate that severe hypothermia can differentially affect the survival of 293T, SaOS2 and Hela cells. Although these cell lines had distinctive response to severe hypothermia, they all maintained a high level of viability under mild hypothermia, suggesting that the mild hypothermia can improve the survival status of mammalian cells in our tested system.

### The effect of hypothermia on the viabilities of suspension, primary and stem cells

We have so far determined the effect of hypothermia on the survival of several transformed cell lines, but all of them are adherent cell lines. Whether the observations from adherent cell lines can be reproduced in suspension, primary and stem cells remains unclear. To address this question, suspension cells (K562) were used to examine the effect of hypothermia on cell survival using the same strategies as done in adherent cell lines. Fig 3A and 3B show that K652 cells stored at 1°C for 2 days showed a high level of death compared with the cells at 16°Cor 22°C. However, the survival rate at different temperatures showed little difference under incubation for 24 hours (Fig 3B); apparently, K562 cells had less sensitivity to the temperature of 5°C than that of 1°C. The effect of hypothermia on K562 cells quite resembled that on Hela cells (Figs 2E and 3B). We next examined the effect of hypothermia on the survival of primary cells and mouse ES cells. Unexpectedly, the survival rate for mouse embryo fibroblast (MEF) cells wasn’t much affected by hypothermic treatment for 3 days; but significantly decreased when stored at 1°C for 4 days (Fig 3C). The result from mouse ES assay show that incubation at 1°C or 5°C significantly affected the survival rate for ES cells when compared with that at 16°C or 22°C (Fig 3D and S2 Fig); the number of live cells rapidly reduced when stored at 1°C or 5°C for 24 hours. Furthermore, the viability for ES cells can decrease to 40–50% at the fourth day under mild hypothermia; suggesting that stem cells are more sensitive to hypothermia than transformed cell lines. Colony formation assays confirmed that preservation of ES cells at 1°C or 5°C caused a poorer recovery than that at 16°Cor 22°C (Fig 3E). Taken together, these data confirm that severe hypothermia indeed affects cell survival although the sensitivities of different cell types to hypothermia showed distinct.

All of the cell lines tested above were stored in their own culture medium; we next examined whether mammalian cells can survive longer when stored in commercial preservation medium under hypothermia. To this end, M2 cells was initially stored in DMSO and serum-free cryopreservation medium (Sigma) at 1°C or 22°C. Strikingly, most of cells have been dead after stored at 1°C for 24 hours or 22°C for 72 hours (S3A Fig); suggesting that cryopreservation medium is more harmful to M2 cells than its own culture medium under hypothermia. It has been described that Leibovitz’s L15 medium can preserve blood cells and maintain their clonogenic capacity for 7 days [16]. Therefore, M2 and Hela cells were preserved in complete L15 medium under hypothermia, the result shows that L15 medium did not improve cell survival rate compared with their own culture mediums (S3B–S3E Fig, Figs 1D and 2E); however, mild hypothermia did enhance the viability for these cell lines compared with severe hypothermia.

### M2 Cell death is mainly caused by apoptosis under hypothermia

**Fig 4:**
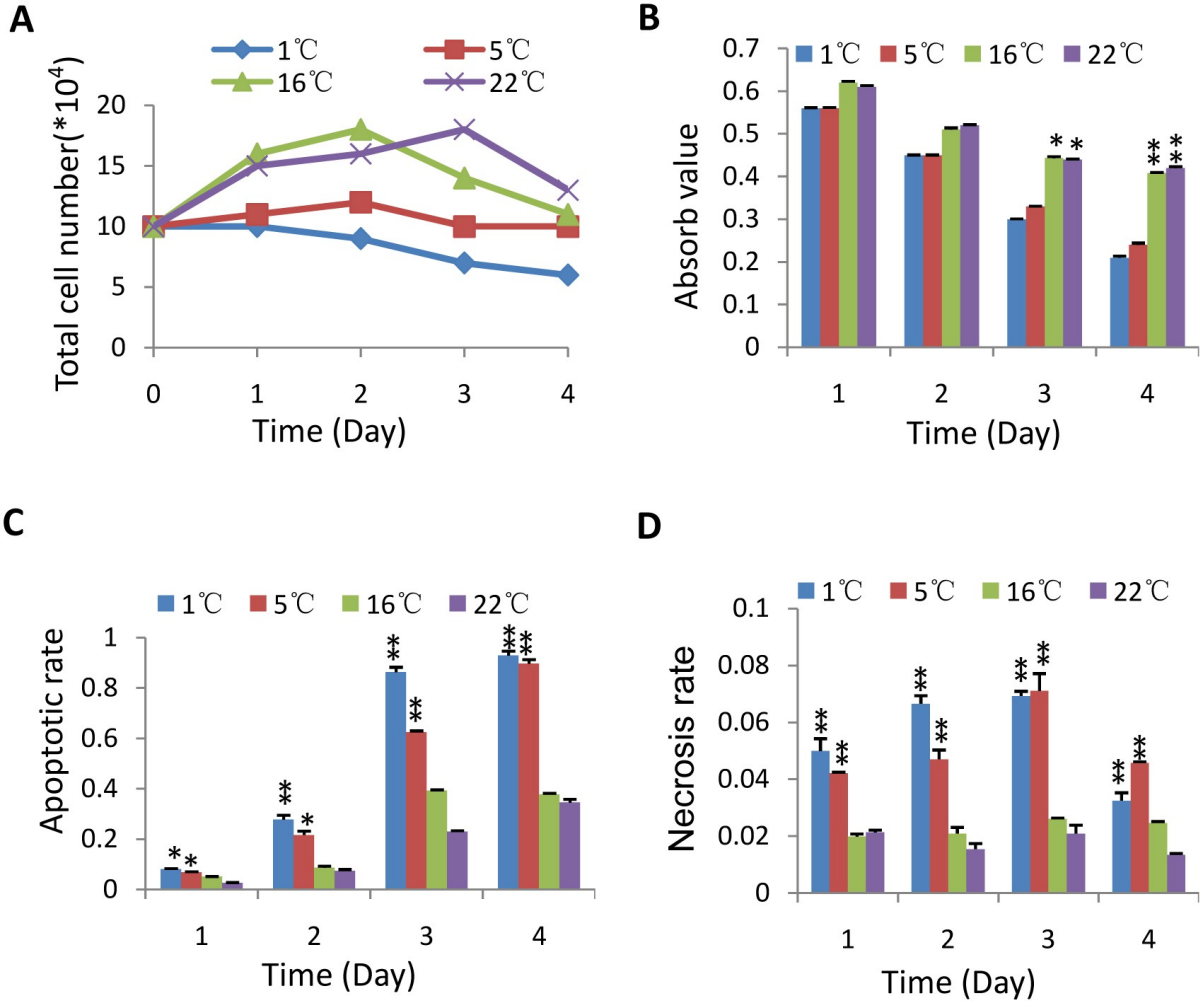
M2 cell death is mainly caused by apoptosis under hypothermia. A) Proliferation analysis for M2 cells treated at indicated temperatures. The total number of treated cells were counted under white light microscope at different time after hypothermic treatment and subjected to statistical analysis. B) MTT assays for M2 cells treated at indicated temperatures. C and D) Apoptotic and necrotic analyses for M2 cells treated under hypotherima. Each bar in B-D represents the mean of three independent experiments with standard deviation (SD). Significant difference was analyzed by comparing the values at different temperatures. *represents P<0.05, ** represents P<0.01, P value was obtained by student’s *t* test.

To understand how mild hypothermia maintains cell survival, cell proliferation assays were performed using M2 cells. The data show that the total cell number including live and dead cells increased up to 65% at the first three days, but decreased to the original level at the fourth day when they were stored at 22°C (Fig 4A). The cell number started to decrease when stored at 1°C after 24 hours, but showed little change during the storage at 5°C. These data suggest that M2 cells remained proliferation at some extent under mild hypothermia. MTT assays illustrated that cell metabolic activity gradually decreased with the time delay during incubation, but significantly reduced at 1°C or 5°C after 48 hours compared to that at 16°C or 22°C (Fig 4B). These results are consistent with those from FDA and PI assays. We next sought to understand how severe hypothermia affects the cell activity using apoptotic/necrotic assays. Fig 4C shows that apoptotic rate increased as the time prolonged, however, the apoptotic rate at 1°C or 5°C was significantly higher than that at 16°C or 22°C. Necrotic rate remained low level for all of the treatments although they showed distinct between severe hypothermia and mild hypothermia (Fig 4D), indicating that M2 cell death under hypothermia is mainly caused by apoptosis rather than necrosis.

### Hypothermic treatment does not change the morphology of mammalian cells and the identity of stem cells

**Fig 5:**
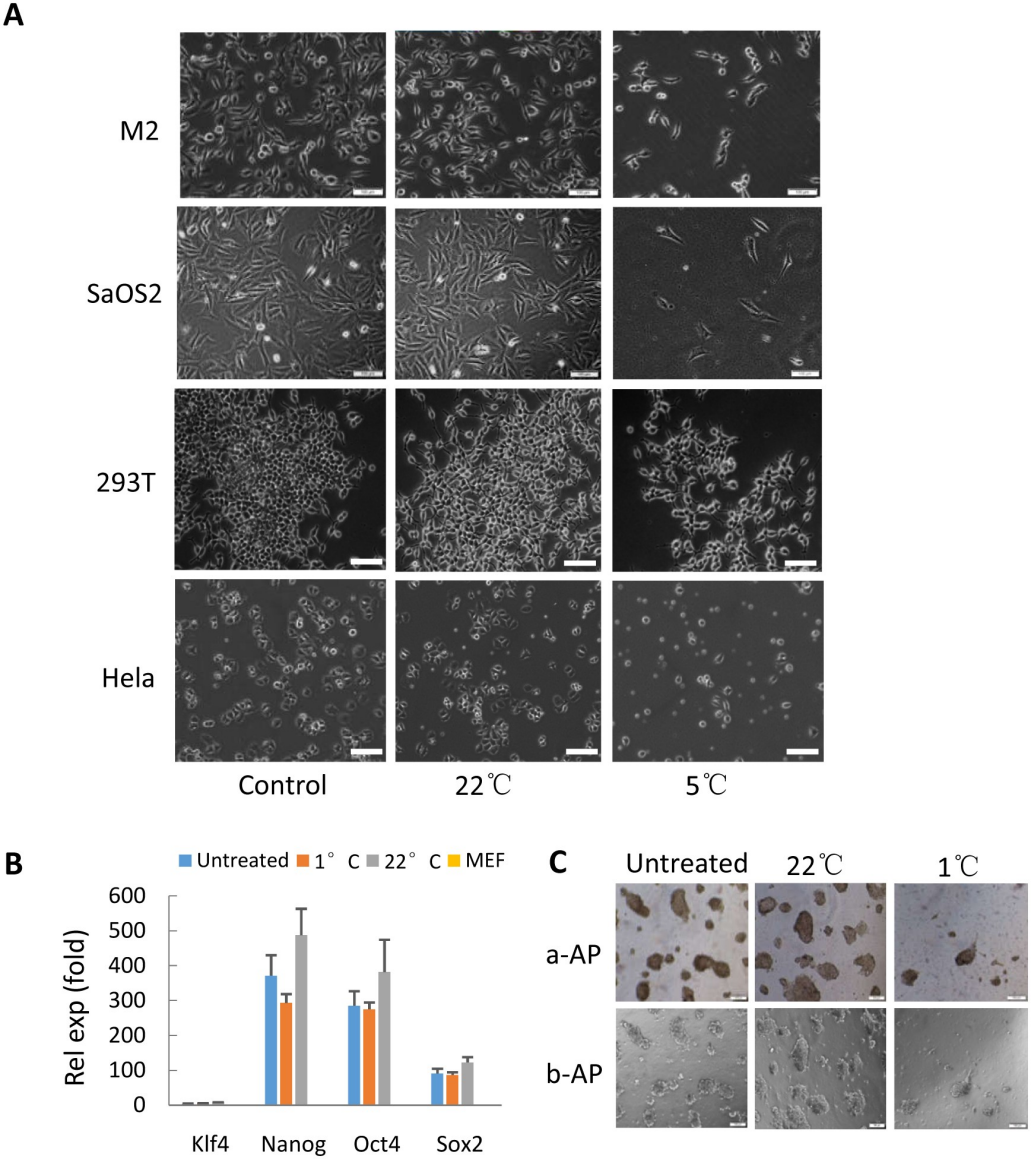
The hypothermic treatment does not change the morphology of transformed cell lines and the identity of stem cells. A) The morphological analysis of transformed cell lines stored at 1°Cor 22°C. Transformed cell lines were incubated at 1°C or 22°C for 48 hours, equal number of the cells was seeded in 6-well plates. After growing for 48 hours, the cells were imaged under bright-field microscope. B) RT-qPCR showing the gene expression of stem cell marker in mouse fibroblast cells (MEF) and in treated and untreated mouse ES cells. C) Alkaline phosphatase (AP) staining for the treated and untreated mouse ES cells. The scale bars in A and C represent 50 μm.

When cells are suspended in culture medium and stored under hypothermia, the stress will be gradually given rise to the cells, and might affect the physiological status of the cells. We next determined whether the cold stress changes the morphology of mammalian cells. To this end, several adherent cell lines and mouse stem cell line were incubated for 48 hours at 1°C or 22°C. The treated cells were seeded in 12-well plates and grew for 2 days; then imaged under bright-field microscope. Fig 5A shows that the cell shape for all of the detected cell lines exhibited little change between the treated and untreated cells, indicating that hypothermic treatment does not change the morphology of mammalian cells. Since stem cell is very sensitive to environmental stimuli, we next determined whether the treatment of hypothermia affects the identity of stem cells. Mouse ES cells were respectively stored at 1°C and 22°C for 3 days and re-cultured in feeder-free condition for 2 days. The cells were then used for analyzing gene expression for stem cell markers. Fig 5B illustrates that the ES cells stored under hypothermia didn’t change gene expression for stem cell markers. Another hallmark of stem cell is to express alkaline phorsphatase (AP); thus, the treated stem cells were used for colony formation assay and detection for AP expression. Fig 5C confirms that the treated stem cells indeed expressed alkaline phosphatase as did the untreated cells; indicating that hypothermic treatment does not affect the identity of stem cells.

### Mammalian cells can maintain a high level of viability after shipment for long distance at ambient temperature

**Fig 6:**
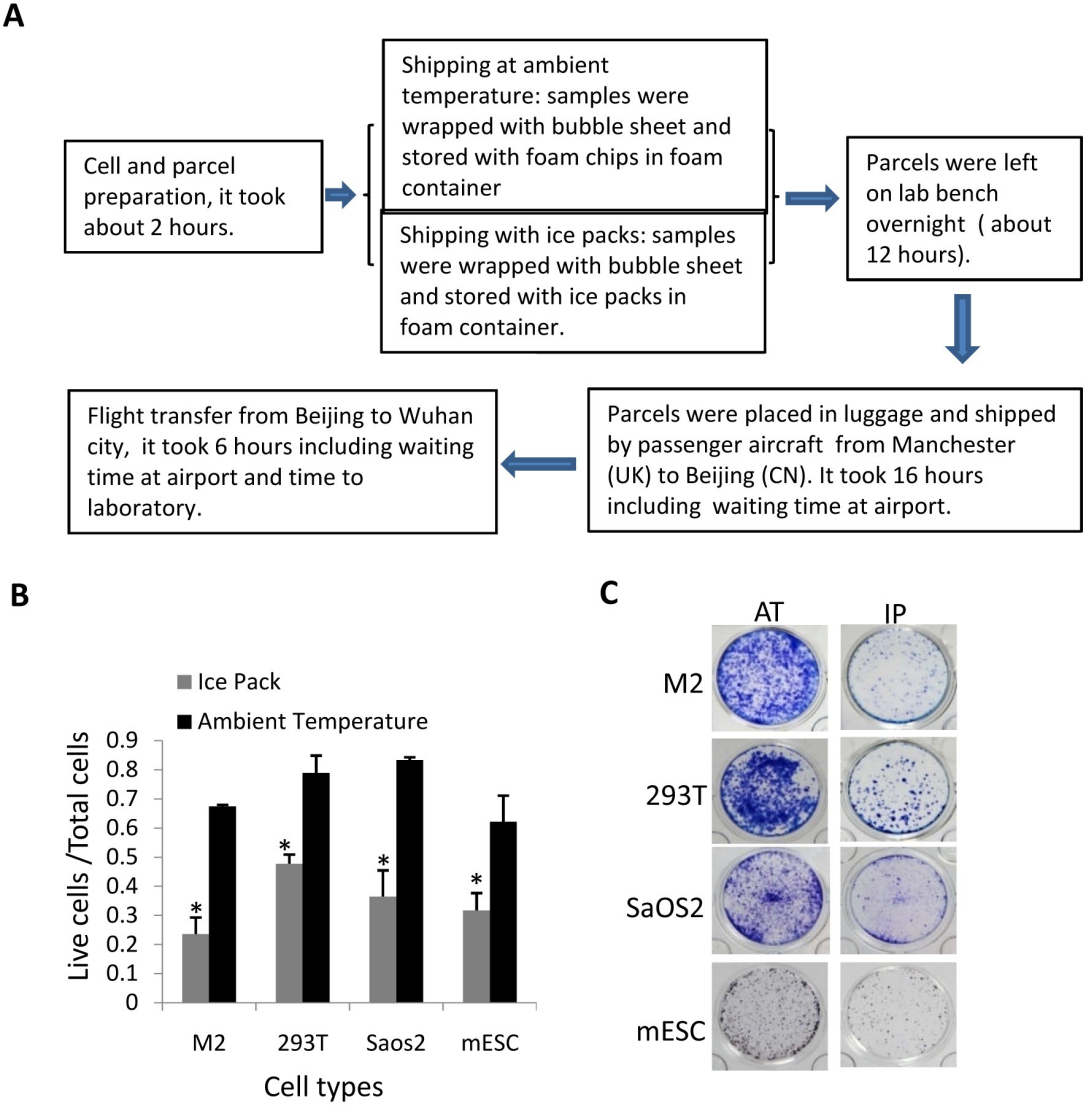
Mammalian cell lines maintain a high level of viability after shipment for long distance at ambient temperature. A) A scheme showing how mammalian cell lines were treated, packed and transported as well as the time consumed. B) The effect of shipment method on the viabilities of mammalian cell lines. Mammalian cells were treated and transported as described in A. At the destination, the viabilities for mammalian cell lines were analyzed as Fig 1D. Each bar represents of the mean of triplicate samples with standard deviation. Significant difference between treatments was analyzed as in Fig 1D. *represents P<0.05 P value was obtained by student *t* test. C) Colony formation analysis for mammalian cell lines. Mammalian cell lines were treated and transported as described in A. At the destination, the cells were used for colony formation assays as in Fig 1E.

As described above, mammalian cell lines incubated at 16°C or 22°C for 4 days still maintained a high level of viability (over 80%), suggesting that mammalian cells could be transported for long distance at environmental temperature. To verify the hypothesis, a case study was performed by transporting three transformed cell lines and a stem cell line from UK to China. These cell lines were shipped with two different methods based on the above findings. The cells were directly suspended in culture medium, followed by packaging and transportation as described in Fig 6A. After transported for 36 hours, the cells were used for viability analysis and colony formation assays. Fig 6B demonstrated that the viabilities for M2, 293T and SaOS2 cells transported at environmental temperature reached up to 80%;but the survival rate of stem cells was round 60%, which is lower than that of transformed cell lines, confirming that stem cells are more sensitive to ambient temperature than transformed cells (Figs 3D and 6B). However, shipment with ice packs significantly reduced the viabilities of all cell lines compared with that at environmental temperature, suggesting that mammalian cells are not suitable for shipment with ice packs. Colony formation assays confirm that the cells shipped at environmental temperature showed better recovery than they shipped with ice packs (Fig 6C). These results indicate that mammalian cells can maintain a high level of viability and recovery after shipment for long distance at ambient temperature.

## Discussion

It is well-known that temperature is one of the most important factors for mammalian cell survival *in vitro*. Temperatures such as 1°C and 4°C or 5°C are often used to deal with the cells in the laboratory. In this study, we show that mammalian cells stored at 1°C or 5°C significantly reduced the viability and recovery although their viabilities are distinguished under the treatment of hypothermia (Figs 1E and 2B–2D). It has been shown that short-term storage of hematopoietic cells at 4°C substantially reduces the live cell number and function of progenitors after stored for 3 days [17], however, Louis et al showed that short-term processing at 4°C before cryopreservation can improve cell recovery compared with that at room temperature [18]. Our result is agreement with the former’s when mammalian cell lines were directly suspended their own culture medium and stored under severe hypothermia. This result is quite unexpected and would have profound influence on laboratory approaches; for instance, the cells are often incubated on ice either before lysing or cell sorting. The results from our study suggest that mammalian cells seem not suitable for incubation on ice or storage at 4 or 5°C, particularly for the cells that are sensitive to severe hypothermia or that need further culturing; thus, our finding extends the understanding on the effect of severe hypothemia on cell survival. IT has been described that hypothermic damage to cell can be improved by the utilization of conservation medium [16,19,20]. In this study the leibovitz’s L15 medium was used to store M2 and Hela cells under hypothermia, but no improvement had been observed for the viability. Previous work showed that the ratio of oxygen and carbon dioxide within storage container can affect cell viability when blood cells were stored at 4°C [17, 21, 22]; however, how the ratio of oxygen and carbon dioxide affect the viability of mammalian cell lines within our tested system remains to be investigated in the future.

Mononuclear cells can be obtained a higher yield from the blood cells shipped at 22°C when compared with them shipped under environmental temperature of 40°C [15]. The preservation of endothelial cells at 10°C can reduce oxidative stress and cold-induced injury [23]. In our work mammalian cells were able to maintain a considerably high level of viability and excellent recovery when stored at 16°C or 22°C for 4 days (Figs 1D and 2B–2D). This observation is likely ascribed to subsequent cell proliferation and reduced apoptotic rate (Fig 4). Our data suggest that mammalian cells could be directly suspended in culture medium and shipped for long distance at ambient temperature. Indeed, a case study confirmed that mammalian cells were able to maintain a high level of viability and recovery after transported for 36 hours from UK to China at environmental temperature (Fig 6B and 6C); whereas the cells shipped with ice pack had poor viability and recovery, suggesting mammalian cell lines are not suitable to be transported with ice pack for long distance when directly suspended culture medium. In addition, it was also observed that 293T cells could maintain a high level of viability after incubation at 37°C for 3 days (data no shown), suggesting that mammalian cell can also be transported under a wide-range of environmental temperature as long as the concentration of cells is optimal.

Mammalian cells can be transported for long distance at ambient temperature when the cells are mixed with agarose- or matrigel-based media [2, 6]. Here we demonstrated that mammalian cells can also be shipped for long distance at ambient temperature when the cells were directly suspended in culture medium. Our novel method is simple, practical and low cost because specific equipment and commercial medium aren’t required. It can be applied to cell shipment for short and long distance between laboratories on general purpose. However, it should be cautious for the shipment on clinical purpose where specific equipments and techniques are highly demanded [24]. Taken together, our study not only broadens the understanding to the effect of hypothermia on cell survival, but also provides an alternative approach for cell shipment. Our approach for cell shipment would be beneficial to numerous laboratories in biological and biochemical research fields.

## Supporting information

S1 Fig Hypothermic treatment reduced the viability of M2 cells. (PDF)

S2 Fig Fluorescence microscopy showing the live and dead cells for mouse embryo stem cells stored under hypothermia. (PDF)

S3 Fig Treatment with preservation medium did not improve the viability compared to that with their own medium. A) Viability analysis for M2 cells that stored in DMSO and serum-free preservation medium at 1°C or 22°C. B and C) Viability analysis for M2 cells that stored in Leibovitz’s L15 medium under hypothermia. The treated cells stained by FDA and PI staining and followed by imaging with fluorescent microscope (B) and statistical analysis (C). D and E) Viability analysis for Hela cells that stored in Leibovitz’sL15 medium under hypothermia. The treated cells stained by FDA and PI staining and followed by imaging with fluorescent microscope (D) and statistical analysis (E). The scale bars in B and D represent 50 micrometer. In A, C and E, each bar represents the mean of three independent experiment with standard deviation (SD). Significant difference was analyzed by comparing the value of the sample at 1°C with that at other temperatures respectively. *represents P<0.05, ** represents P<0.01, P value was obtained by student’s *t* test.
(PDF)
