# Supplementary figures and images for: The analysis of viability for mammalian cells treated at different temperatures and its application in cell shipment
Source: PLoS One. 2017 Apr 18;12(4):e0176120. doi: 10.1371/journal.pone.0176120 (PMC5395231; doi:10.1371/journal.pone.0176120)

**S1Fig**

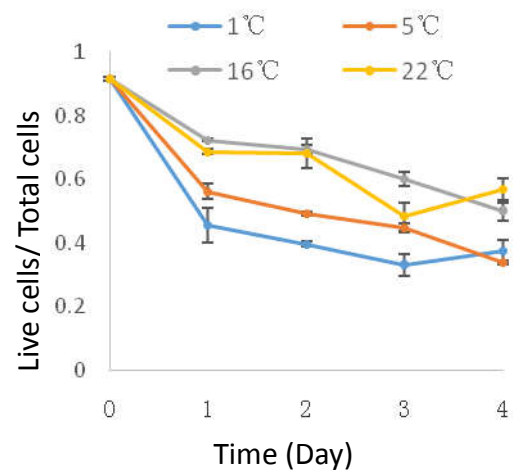

Supplement: S1 Fig — (PDF) [file pone.0176120.s001.pdf]

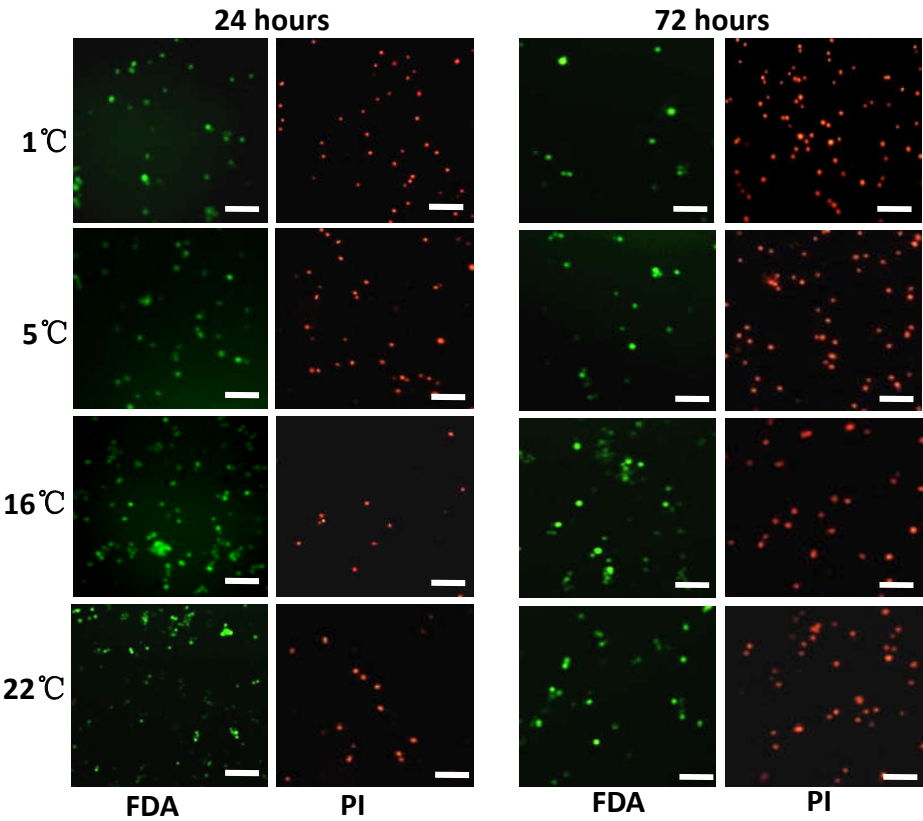

Supplement: S2 Fig — (PDF) [file pone.0176120.s002.pdf]

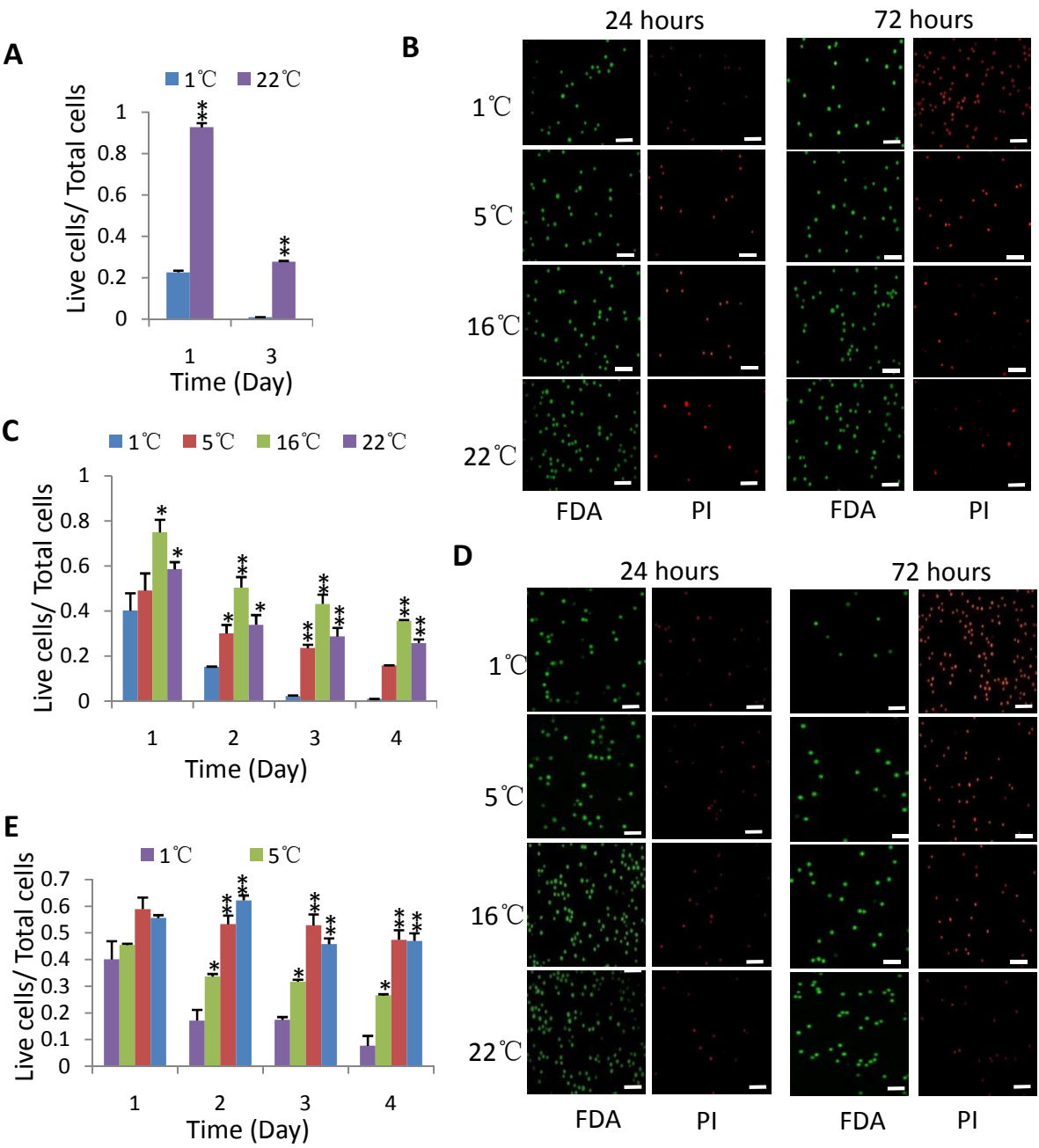

Supplement: S3 Fig — A) Viability analysis for M2 cells that stored in DMSO and serum-free preservation medium at 1°C or 22°C. B and C) Viability analysis for M2 cells that stored in Leibovitz’s L15 medium under hypothermia. The treated cells stained by FDA and PI staining and followed by imaging with fluorescent microscope (B) and statistical analysis (C). D and E) Viability analysis for Hela cells that stored in Leibovitz’sL15 medium under hypothermia. The treated cells stained by FDA and PI staining and followed by imaging with fluorescent microscope (D) and statistical analysis (E). The scale bars in B and D represent 50 micrometer. In A, C and E, each bar represents the mean of three independent experiment with standard deviation (SD). Significant difference was analyzed by comparing the value of the sample at 1°C with that at other temperatures respectively. *represents P<0.05, ** represents P<0.01, P value was obtained by student’s t test. (PDF) [file pone.0176120.s003.pdf]
